# When cardiology meets endocrinology: sustained atrial flutter associated with thyrotoxic periodic paralysis

**DOI:** 10.1093/omcr/omac020

**Published:** 2022-03-16

**Authors:** Alejandro Sanchez-Nadales, Valentina Celis-Barreto, Alejandra Diaz-Sierra, Andres Sanchez-Nadales, Antonio Lewis, Jose Sleiman

**Affiliations:** Department of Cardiovascular Disease, Cleveland Clinic Florida, Weston, FL, USA; Department of Medicine, Mount Sinai Medical Center of Florida, Miami Beach, FL, USA; Department of Medicine, Advocate Illinois Masonic Medical Center, Chicago, IL, USA; Department of Medicine, Universidad Central de Venezuela Facultad de Medicina, Caracas, Distrito Capital, Venezuela; Department of Cardiovascular Disease, Cleveland Clinic Florida, Weston, FL, USA; Department of Cardiovascular Disease, Cleveland Clinic Florida, Weston, FL, USA

**Keywords:** atrial flutterthyrotoxic periodic paralysisGraves’ disease

## Abstract

Periodic paralysis is a rare muscle disease that manifests as episodes of painless muscle weakness, and the hypokalemic form is commonly associated with hyperthyroidism. Most tachyarrhythmias related with thyrotoxicosis include sinus tachycardia and atrial fibrillation, but an association between thyrotoxic hypokalemic periodic paralysis and typical atrial flutter has seldomly been documented. Here, we present the case of a young male who was diagnosed with thyrotoxic periodic paralysis causing cavotricuspid isthmus-dependent atrial flutter, successfully treated with diltiazem, propranolol, methimazole, potassium iodine (SSK) and rivaroxaban.

## INTRODUCTION

Periodic paralysis is a rare muscle disease that manifests by painless muscle weakness often precipitated by heavy exercise, fasting or high-carbohydrate meals. It is classified as either hyperkalemic or hypokalemic, with the hypokalemic form being most associated with thyrotoxicosis. Thyrotoxicosis is commonly associated with atrial fibrillation but has less frequently been reported to induce atrial flutter.

## CASE REPORT

**Figure 1:**
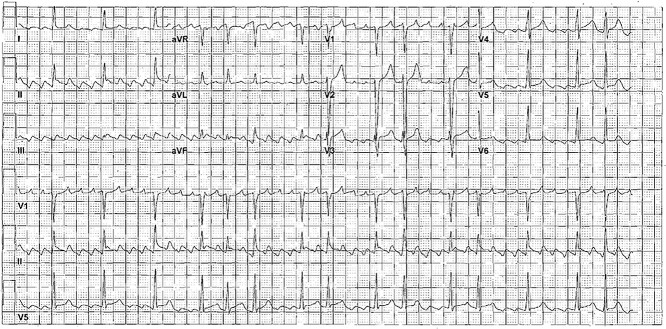
12-lead ECG showed a rapid typical cavotricuspid isthmus-dependent atrial flutter and a rapid ventricular rate. Counterclockwise atrial flutter with flutter waves negative in II, III, aVF and positive waves in V1

A 38-year-old Caucasian male with a history of essential hypertension on amlodipine presented to the hospital complaining of recent onset intermittent lower extremity weakness and palpitations. On examination, he was afebrile with a heart rate of 180 bpm, tachypneic with a respiratory rate of 22 rpm, and hypertensive with a blood pressure of 167/92 mmHg. Neurologic exam revealed paraparesis with lower extremity strength of muscle power assessment (MRC) score 3/5. Initial laboratory workup revealed hypokalemia at 1.7 mEq/L (normal 3.4–5 mEq/L), and the electrocardiogram (ECG) revealed atrial flutter with rapid ventricular response (Fig. 1). He was initially treated with oral metoprolol tartrate and oral diltiazem, but subsequently required initiation of a diltiazem drip at a rate of 15 mg/h. Thyroid laboratory workup revealed a thyroid-stimulating hormone (TSH) level < 0.008 mU/L (normal 0.35–5.0 mU/L), free T4 level > 8 ng/dL (normal 0.8–1.5 ng/dL) and total T3 of 5.23 ng/mL (normal 0.6–1.81 ng/mL). The patient was diagnosed with thyrotoxic periodic paralysis (TPP) based on the thyroid panel results, hypokalemia and clinical presentation.

**Figure 2:**
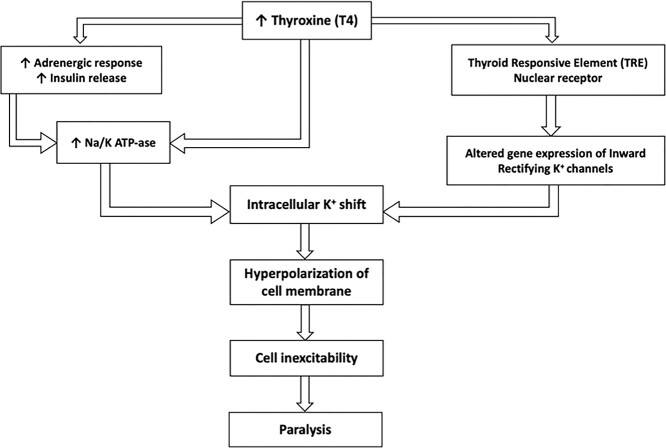
Mechanism of TTP

Based on these findings, propylthiouracil 200 mg every 4 h, prednisone 20 mg twice a day, and SSKI every 6 h were prescribed to manage the thyrotoxic state along with propranolol 160 mg every 8 h for additional reduction of peripheral conversion of T4 to T3. Moreover, therapeutic anticoagulation with rivaroxaban 20 mg daily was initiated regardless of a CHA_2_DS_2−_VASCs score of 0 points as the flutter was deemed to be induced by the thyrotoxicosis.

A transthoracic echocardiogram was performed, revealing a normal left ventricle ejection fraction of 55% and no evidence of significant structural heart disease. Subsequent endocrine workup revealed thyroid-stimulating immunoglobulin (TSI) elevated to 33.4 mU/L (normal 0.4–4.0 mU/L) consistent with the diagnosis of Graves’ disease (GD). Patient recovered normal neurological function with MRC 5/5 after 6 h of treatment initiation.

Therapeutic modalities that included radioactive iodine and surgery were discussed and deferred for the future. SSKI was discontinued after one week of treatment, and he was discharged and advised to continue methimazole for the following 12 months. He spontaneously converted back to normal sinus rhythm 2 weeks after medical discharge without the need for electrical or pharmacological cardioversion after maintaining an adequate control of his hyperthyroidism.

## DISCUSSION

Thyrotoxicosis is most associated with atrial fibrillation since thyroid hormone is known to have arrhythmogenic activity by acting on nuclear thyroid receptors and increasing sympathetic activity. Thyroid hormone upregulates the transcription of cardiac proteins, when acting on nuclear thyroid receptors. Thyroid hormones also exert an upregulating effect on the sarcoplasmic reticulum leading to calcium release and a positive chronotropic/inotropic effect [1]. Most cases relating thyrotoxicosis to tachyarrhythmias usually refer to atrial fibrillation, but sustained atrial flutter rarely occur in these scenarios.

GD, known as the most common cause of hyperthyroidism, is an autoimmune disorder characterized by thyroidal and extrathyroidal manifestations. Graves’ is caused by the formation of activating thyrotropin-receptor antibodies that induce thyroid hormone overproduction. Exacerbation of GD may lead to thyroid storm, a potentially life-threatening endocrine emergency, as well as TPP [2]. TPP is another severe but rare complication with a reported incidence of 0.2% in North America and seen more frequently in males and Asians. It is characterized by muscle paralysis, and acute hypokalemia due to a potassium shift into muscle cells (Fig. 2). Hypokalemic periodic paralysis (HPP) is the variant most seen in patients with GD in association with acute thyrotoxicosis, TTP is a sporadic form [3].

The most common arrhythmias related to TPP and HPP are sinus tachycardia, and first-degree atrioventricular block. However, atrial flutter has not previously been illustrated along these clinical scenarios [3].

When treating HPP, the goal is to address the symptoms caused by a hyperthyroid state and prevent recurrent attacks by controlling possible inducing factors. Despite the nomenclature, potassium reserves are normal in these patients. Supplementation is done to secure normal plasma levels of potassium. Intravenous potassium chloride is the first line of treatment for HPP [4]. In cases that proved resistant to this intervention, intravenous propranolol effectively managed the paralytic symptoms. The effectiveness of propranolol can be attributed to its effect on the Na+/K+ ATPase [5]. Antithyroid drug management is widely considered as the gold standard of therapy; however, radioiodine ablation is more prevalent as first-line in North America. Nevertheless, the underlying etiologies of hyperthyroidism should be investigated and treated. [5].

Addressing the rhythm disorder can be divided into two steps [6]. The first is managing rate control, which can be achieved using beta-blockers, calcium channel blockers or amiodarone. The comorbidities should be considered when deciding on the agent to use. Propranolol has the advantage of reducing the peripheral conversion of T4 to T3 [5]. Calcium channel blockers should be used with caution since they affect peripheral resistance, and patients with thyrotoxicosis already present with a low systemic vascular resistance.

Amiodarone can be considered since it has a double advantage by reducing peripheral conversion of T4 to T3, inhibiting thyroid hormone synthesis due to its high iodine content, and exerting its antiarrhythmic properties by blocking potassium rectifying currents leading to an increase in action potential duration and a prolonged effective refractory period of the AV node [7]. The combination of properties can result in a more rapid resolution of symptoms in patients with arrhythmia secondary to thyrotoxicosis. On the other hand, amiodarone is rich in iodine and theoretically it may enhance thyrotoxicosis and type 1 amiodarone-induced thyrotoxicosis is more frequent in patients with underlying thyroid diseases such as GD [7]. Thioamides should be given 1 h before SSKI or amiodarone. The second step to consider is preventing the arrhythmia’s maintenance, which can be achieved with the long-term use of the agents discussed above as prophylaxis for recurrent attacks. In this case, propranolol would be the agent of choice.

Lastly, we decided to use prophylactic anticoagulation regardless a CHA_2_DS_2−_VASCs score of 0 points based on disagreement in the medical literature regarding the effectiveness of this strategy in patients with unknown duration of hyperthyroidism states, even more when transesophageal echocardiography is not pursued [8].

GD may lead to potentially life-threatening emergencies, such as thyroid storm or TPP. Cardiac testing is indicated in patients with thyrotoxicosis. The underlying etiologies of hyperthyroidism should be investigated and treated accordingly. Propranolol is the beta-blocker of choice in cases of thyrotoxicosis. TPP can be a trigger for the development of sustained atrial flutter. In our case, appropriate treatment was associated with spontaneous resolution of the atrial flutter without the need for cardioversion or ablative intervention.

## Data Availability

The data supporting the findings of this study are available from the corresponding author upon reasonable request.
